# Genetic variation of lncRNA GAS5 contributes to the development of lung cancer

**DOI:** 10.18632/oncotarget.19955

**Published:** 2017-08-03

**Authors:** Weihao Li, Kai Huang, Fengbiao Wen, Guanghui Cui, Haizhou Guo, Song Zhao

**Affiliations:** ^1^ Department of Thoracic Surgery, The First Affiliated Hospital of Zhengzhou University, Zhengzhou, Henan 450052, P.R. China; ^2^ Department of Oncology, The First Affiliated Hospital of Zhengzhou University, Zhengzhou, Henan 450052, P.R. China

**Keywords:** lung cancer, lncRNA, GAS5, susceptibility

## Abstract

Lung cancer remains the leading cause of cancer-related deaths throughout the world. In spite of great effort for the research of carcinogenesis, the molecular mechanisms of lung cancer remain unclear. In current study, we investigated the possible association between susceptibility of lung cancer and GAS5 rs145204276, which showed contradictory roles in carcinogenesis of colorectal cancer and hepatocellular carcinoma. We found that the del allele was significantly associated with 21% decreased risk of lung cancer (OR=0.79; 95% CI=0.66-0.93; P value = 0.006). Compared with the genotype ins/ins, both the genotype ins/del (OR=0.78; 95% CI=0.62-0.99) and del/del (OR=0.59;95% CI=0.39-0.89) showed decreased susceptibility of lung cancer. Real-time PCR analysis found that the expression levels of lncRNA GAS5 in lung cancer tissues were significantly lower than those in the corresponding normal tissues (P<0.01). Also the relative GAS5 expression level in samples with del/del genotype was significantly higher than that in samples with ins/del and ins/ins genotype (P<0.01). Taken together, our findings provided strong evidence for the hypothesis that GAS5 rs145204276 were significantly associated with the susceptibility of lung cancer, and GAS5 functions as a tumor suppressor in carcinogenesis of lung cancer.

## INTRODUCTION

Lung cancer ranks the leading cause of cancer death among males in both more and less developed countries [1–3]. Although rates are now decreasing in most of these countries, they are increasing in countries where smoking uptake occurred later (e.g., China) [1, 2, 4]. According to the report of National Office for Cancer Prevention and Control of China, the estimated annual new lung cancer cases was 733.3 thousands, and the new deaths was 610.2 thousands [5]. This mainly caused the increasing smoking rate, which has been defined as one of the behavioral risk factors for many chronic diseases [6, 7]. To date, in spite of great effort for the research of carcinogenesis, the molecular mechanisms of lung cancer remain unclear.

Recently, the dysregulation of long non-coding RNAs (lncRNAs), which are longer than 200 nucleotides and cannot be translated, has been identified in a variety of cancers due to their critical role in the regulation of cellular processes [8–10]. The lncRNA growth arrest special 5 (GAS5), a pivotal tumor suppressor long noncoding RNA in human cancers, has been identified to be involved in carcinogenesis progress of several cancers, including breast cancer, prostate cancer, hepatocellular carcinoma, and colorectal cancer [11–13]. Tao et al identified that GAS5 rs145204276 could increase the expression of GAS5, and then the risk of hepatocellular carcinoma [13]. However, Zheng et al found that the allele del of rs145204276 was significantly associated with 21% decreased risk of colorectal cancer. These finding raised the concern of the differential roles of lncRNA GAS5 in carcinogenesis of different cancer types. To clarify the potential role of GAS5 rs145204276, a deletion allele of a 5-base pair del polymorphism in the promoter region of GAS5, we conducted this case-control study among a Chinese population, and investigated the possible association between GAS5 rs145204276 and susceptibility of lung cancer.

## RESULTS

### Characteristic of the study population

The distribution of selected characteristics between lung cancer patients and control subjects is shown in Table 1. In total, there were 600 cases and 600 controls included in this case control study. No significant differences were observed between cases and controls for age (P = 0.620) and gender (P = 0.691), which indicates satisfactory matching of controls by age and gender. While a significant difference were observed for the distribution of Smoking status (**P<0.001**), which reveals lung cancer patients are more likely to be smokers.

**Table 1:** Distributions of selected variables in lung cancer cases and cancer-free controls

	Cases (n=600)	Controls (n=600)	P value
Age			
<60	239 (39.8%)	231 (38.5%)	0.620
≥60	361 (60.2%)	369 (61.5%)	
Gender			
Male	425 (70.8%)	419 (69.8%)	0.691
female	175 (29.2%)	181 (30.2%)	
Smoking status			
Ever	218 (36.3%)	126 (21.0%)	**P<0.001**
Never	382 (63.2%)	474 (79.0%)	
**Histology**			
*Adenocarcinoma*	231 (38.5%)		
*Squamous cell*	369 (61.5%)		

### The associations of GAS5 rs145204276 with lung cancer susceptibility

The observed genotypic frequencies of rs145204276 in the controls were in Hardy-Weinberg equilibrium (P > 0.05). Table 2 presents the association between GAS5 rs145204276 and susceptibility of lung cancer. The allele del was significantly associated with 21% decreased risk of lung cancer (OR=0.79; 95% CI=0.66-0.93; P value = 0.006).

**Table 2:** Associations between GAS5 rs145204276 and lung cancer susceptibility

Genetic model	Genotype	Cases	Controls	OR (95% CI)^a^	P value
Codominant model	ins/ins	287	246	1.00 (Reference)	
	ins/del	270	292	0.78 (0.62-0.99)	**0.044**
	del/del	43	62	0.59 (0.39-0.89)	**0.011**
Dominant model	ins/ins	287	246	1.00 (Reference)	
	ins/del + del/del	313	354	0.75 (0.60-0.984)	**0.012**
Recessive model	ins/ins + ins/del	557	538	1.00 (Reference)	
	del/del	43	62	0.66 (0.45-0.98)	**0.042**
Additive model	ins allele			1.00 (Reference)	
	del allele			0.79 (0.66-0.93)	**0.006**

^a^ Adjusted for age, gender, and smoking status.

Compared with the genotype ins/ins, both the genotype ins/del (OR=0.78; 95% CI=0.62-0.99) and del/del (OR=0.59;95% CI=0.39-0.89) showed decreased susceptibility of lung cancer. When analyzed in both dominant and recessive model, the results were still significant.

### Relative expression of GAS5

**Figure 1:**
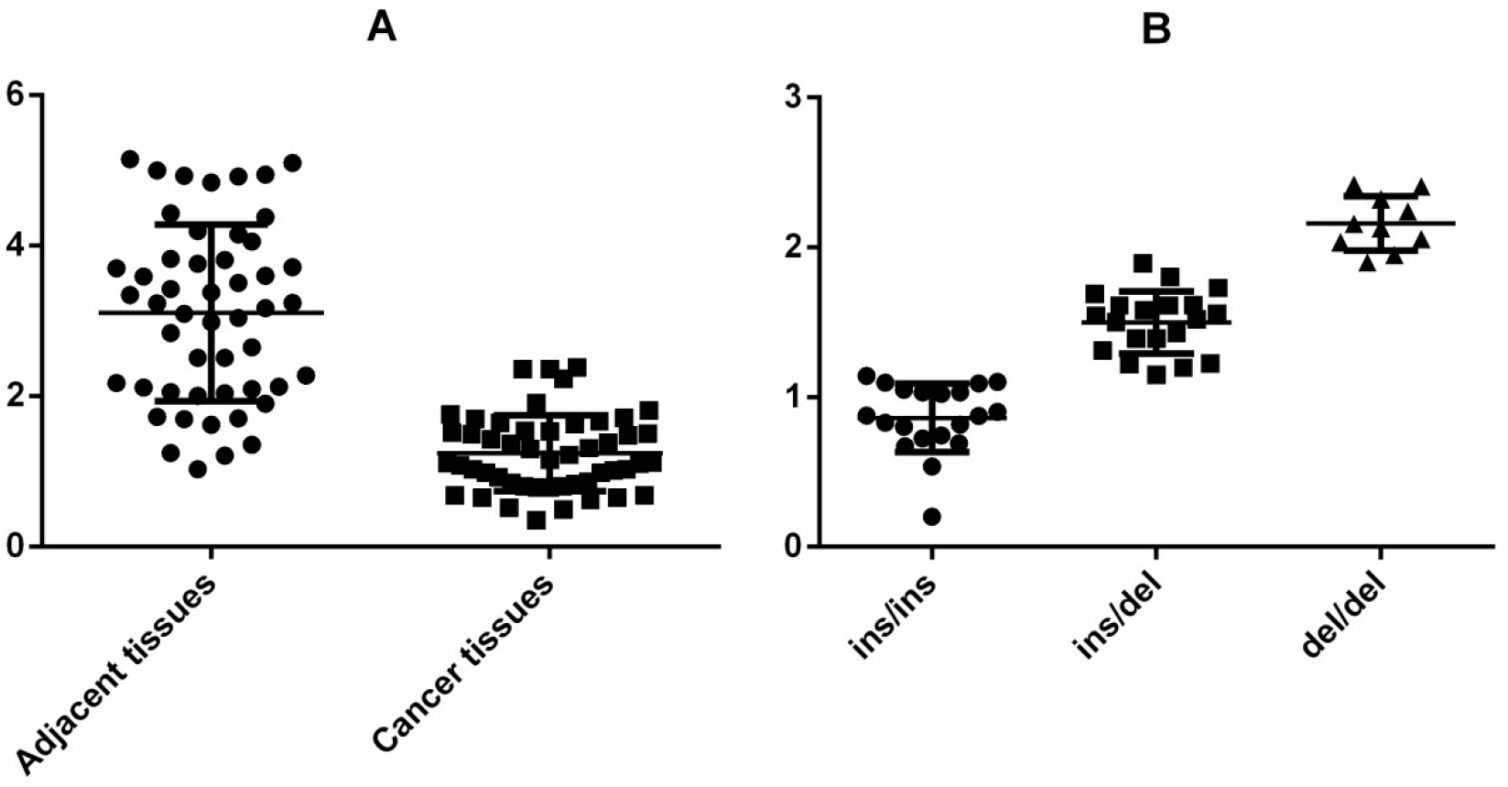
Relative expression of GAS5

Next, we further examined the expression of GAS5 in lung cancer and adjacent tissue samples with different genotypes. As shown in Figure 1, the expression levels of lncRNA GAS5 in lung cancer tissues were significantly lower than those in the corresponding normal tissues (P<0.01, Figure 1A). Also the relative GAS5 expression level in samples with del/del genotype was significantly higher than that in samples with ins/del and ins/ins genotype (P<0.01, Figure 1B).

## DISCUSSION

Increasing evidence revealed that abnormal expression of GAS5 is involved in many kinds of cancers [14]. The circulating GAS5 could be used as a novel biomarker for the diagnosis of non-small cell lung cancer [15]. The current study explored association between GAS5 rs145204276 and susceptibility of lung cancer using a case-control study in a Chinese population. We found the allele del was significantly associated with 21% decreased risk of lung cancer (OR=0.79; 95% CI=0.66-0.93; P value = 0.006). To the best of our knowledge, this should be first study which evaluated the genetic association of GAS5 with the susceptibility of lung cancer.

GAS5, whose transcriptional unit is divided into 12 exons that span around 7 kb, was first isolated from mouse genomic DNA by Coccia et al in 1992 [16]. Then Fleming et al detected it was expressed in the mouse preimplantation embryo [17]. Mourtada-Maarabouni et al first identified that GAS5 transcripts were subject to complex post-transcriptional processing, could controls apoptosis and was down regulated in breast cancer [18]. Furthermore, Liu et al found that down regulation of GAS5 promotes bladder cancer cell proliferation, partly by regulating CDK6 [19]. Lu et al also revealed that down regulation of gas5 increases pancreatic cancer cell proliferation by regulating CDK6 [20]. Subsequently, gas5 was identified as a tumor suppressor, and prognosis marker in renal cell carcinoma, cervical cancer, malignant pleural mesothelioma, gastric cancer, colorectal cancer, endometrial cancer, and breast cancer [11, 21–29]. Mazar, J et al [30] found differentially regulates cell cycle arrest and apoptosis through activation of BRCA1 and p53 in human neuroblastoma. However, only two studies evaluated associations of the genetic variation of lncRNA GAS5 with cancers (colorectal cancer and hepatocellular carcinoma), which raised the concern of the differential roles of lncRNA GAS5 in the carcinogenesis progress of different cancer types [10, 13].

In current study, we found the del allele of GAS5 rs145204276 was significantly associated with decreased risk of lung cancer with a statistical power of 77%. Combined with the results of real-time PCR analysis that the expression levels of lncRNA GAS5 in lung cancer tissues were significantly lower than those in the corresponding normal tissues and the relative GAS5 expression level in samples with del/del genotype was significantly higher than that in samples with ins/del and ins/ins genotype, we can conclude that GAS5 functions as a tumor suppressor in lung cancer, and rs145204276 could increase the expression of lncRNA GAS5 in tissues. These findings were consistent with results of Zheng et al in colorectal cancer, which also showed that the carriers of allele del are less likely to get lymph node metastasis [10].

Our study had its own advantages. First, the moderate sample size was enrolled in current study; second, we characterized the function of SNP rs145204276, making the association of this SNP with the risk of lung cancer biological plausible. Despite of these strengths mentioned above, some limitations should be noted. Limited information of further evaluation of gene–environment interactions, deficiency of independent replication, and unavoidable selection bias for case-control study. However, we still provide strong evidence for the tumor suppressor role of lncRNA GAS5. In summary, we demonstrated that SNP rs145204276 of GAS5was associated with decreased risk of lung cancer in the Chinese populations. These findings should be verified by larger, well-designed epidemiologic and functional studies.

## PATIENTS AND METHODS

### Study subjects

In current study, we enrolled 600 incident cases of lung cancer, and 600 healthy controls, which were matched by age and gender. The diagnosis of patients was validated through pathologic examination by two different senior pathologists, while healthy controls free of any type cancers were selected from the neighboring area with the similar life, diet habits, and environmental exposure. After signing informed consent, we collected 5 ml venous blood from each patient and took a face-to-face interview with a standard questionnaire including information on demographic data.

### DNA extraction and genotyping

we extracted genomic DNA from peripheral blood using phenol chloroform extraction. DNA fragments containing the indel polymorphism were amplified using genotyping primers: F-TCCCGACTGAGGAGGAAGAGCA; R-AACACCGTCCCGGAAGTGAAA. Participants’ status was unrevealed in the genotyping process. For quality control, two duplicated samples were included in each 384-plate for the quality control. The assays were repeated for 5% of the samples, and the results were 100% concordant.

### Real-time PCR analysis

Total RNA from lung cancer and adjacent tissue samples with different genotypes was extracted according to the manufacturer’s protocol (Invitrogen, Carlsbad, CA, USA). SYBR Green real-time PCR (reverse transcriptase-PCR) was performed on Roche Light Cycler 480 system to quantify relative GAS5 expression in these samples. Glyceraldehyde 3-phosphate dehydrogenase (GAPDH) was used as an internal control. The assay was conducted using the ABI 7300 system (Applied Biosystems). The 2^-ΔΔCT^ algorithm was applied to calculate the expression levels for different groups.

### Statistical analysis

All the statistical analyses were two-sided and were performed with the SAS 9.2 software (SAS Institute, Cary, NC, USA), while P value < 0.05 was considered statistically significant. The associations between GAS5 rs145204276 and risk of lung cancer were evaluated by odds ratios (ORs) and their 95% confidence intervals (CIs) from unconditional logistic regression analyses. Chi-square (χ2) test was used to estimate the distribution differences of demographic characteristics as well as genotypes and alleles of the rs145204276 between cases and controls. All ORs were adjusted for age, gender, and smoking status. Hardy–Weinberg equilibrium (HWE) was tested using a goodness-of-ft χ2-test.
